# Right ventricular pacing for hypertrophic obstructive cardiomyopathy: meta-analysis and meta-regression of clinical trials

**DOI:** 10.1093/ehjqcco/qcz006

**Published:** 2019-01-31

**Authors:** Ahran D Arnold, James P Howard, Kayla Chiew, William J Kerrigan, Felicity de Vere, Hannah T Johns, Leonid Churlilov, Yousif Ahmad, Daniel Keene, Matthew J Shun-Shin, Graham D Cole, Prapa Kanagaratnam, S M Afzal Sohaib, Amanda Varnava, Darrel P Francis, Zachary I Whinnett

**Affiliations:** 1 National Heart and Lung Institute, Imperial College London, Hammersmith Hospital, Du Cane Road, London, UK; 2 Cardiology Department, Imperial College Healthcare NHS Trust, Du Cane Road, London, UK; 3 University of Melbourne, Burgundy Street, Heidelberg, Victoria, Australia; 4 Cardiology Department, St Bartholomew’s Hospital, W Smithfield, London, UK

**Keywords:** Hypertrophic cardiomyopathy, Right ventricular pacing, Meta-analysis

## Abstract

**Aims:**

Right ventricular pacing for left ventricular outflow tract gradient reduction in hypertrophic obstructive cardiomyopathy remains controversial. We undertook a meta-analysis for echocardiographic and functional outcomes.

**Methods and results:**

Thirty-four studies comprising 1135 patients met eligibility criteria. In the four blinded randomized controlled trials (RCTs), pacing reduced gradient by 35% [95% confidence interval (CI) 23.2–46.9, *P* < 0.0001], but there was only a trend towards improved New York Heart Association (NYHA) class [odds ratio (OR) 1.82, CI 0.96–3.44; *P* = 0.066]. The unblinded observational studies reported a 54.3% (CI 44.1–64.6, *P* < 0.0001) reduction in gradient, which was a 18.6% greater reduction than the RCTs (*P* = 0.0351 for difference between study designs). Observational studies reported an effect on unblinded NYHA class at an OR of 8.39 (CI 4.39–16.04, *P* < 0.0001), 450% larger than the OR in RCTs (*P* = 0.0042 for difference between study designs). Across all studies, the gradient progressively decreased at longer follow durations, by 5.2% per month (CI 2.5–7.9, *P* = 0.0001).

**Conclusion:**

Right ventricular pacing reduces gradient in blinded RCTs. There is a non-significant trend to reduction in NYHA class. The bias in assessment of NYHA class in observational studies appears to be more than twice as large as any genuine treatment effect.

## Introduction

Hypertrophic cardiomyopathy (HCM) is the most common cause of sudden death in young adults with a prevalence of 0.2%.^1^ When asymmetrical septal hypertrophy leads to left ventricular outflow tract obstruction (LVOTO) with associated systolic anterior motion of the anterior mitral valve leaflet, this confers a diagnosis of the sub-phenotype hypertrophic obstructive cardiomyopathy (HOCM, also known as ‘obstructed HCM’), which occurs in around 70% of HCM sufferers.^1^ Alongside contributing to mortality through multiple mechanisms, including heart failure and malignant arrhythmia, LVOTO produces significant morbidity, inducing symptoms of chest pain, breathlessness, exertion intolerance, light-headedness, and syncope.

Management of symptomatic LVOTO is initially pharmacological but interventions are available in the form of surgical septal myectomy or percutaneous alcohol septal ablation, both carrying risks of complication. Failure, intolerance and reluctance with pharmacological and interventional treatment of LVOTO led to interest in the use of right ventricular pacing as an alternative method for gradient reduction and resultant symptomatic improvement.^2^^,^^3^

A number of studies, both randomized and observational, have investigated the effect of dual chamber pacing in HOCM. We systematically analysed these to quantify the effect on LVOTO, left ventricular systolic function, symptoms and functional status.

## Methods

We carried out a meta-analysis of studies that evaluated right ventricular pacing in HOCM in accordance with PRISMA guidelines.^4^ We prospectively registered this meta-analysis on the PROSPERO international register of systematic reviews (registration number CRD42017062165).^5^

### Search strategy

We searched PubMed and the Cochrane Central Register of Controlled Trials for any studies [randomized controlled trials (RCTs), non-RCTs, and uncontrolled observational studies] published in the English language where adults with HOCM underwent atrial-synchronous right ventricular pacing. Studies were included if prospectively determined clinically relevant outcomes were reported: left ventricular outflow tract gradient (LVOTg), New York Heart Association (NYHA) functional status, left ventricular ejection fraction, exercise duration, and peak oxygen uptake during cardiopulmonary exercise testing. Systematic reviews were examined for references to relevant studies. Any discrepancies were resolved by consensus. The full search strategy is reported in the Supplementary material online.

The study protocol was drafted by A.D.A. and revised by all co-authors. Preliminary search and eligibility analysis was performed by K.C. and Y.A. F.d.V. and A.D.A. performed an independent preliminary search.

### Inclusion and exclusion criteria

We considered all studies of right ventricular pacing in HOCM. Studies were eligible if they recruited patients with an elevated gradient (>30 mmHg).^1^ We excluded case reports, studies of biventricular pacing, or studies where pacing occurred was delivered in combination with other invasive gradient reduction interventions such as septal ablation or myomectomy.

### Endpoints

The primary efficacy endpoint was change in gradient (ΔLVOTg). The secondary efficacy outcomes were change in symptomatic and functional status measured by NYHA class, exercise time, or peak oxygen uptake, from baseline to follow-up. Detailed inclusion criteria for endpoints are found in the Supplementary material online.

### Follow-up

It was anticipated that the search would reveal trials of varying follow-up duration. We prospectively determined that ΔLVOTg would be analysed according to the following groups of mean follow-up durations: immediate (<12 h), short-term (12 h to 6 months), medium-term (>6 months to <2 years), and long-term (>2 years). This would allow testing for progressive change in LVOTg due to remodelling over time.^6^ Randomized studies were pre-specified to be analysed separately from observational studies with comparison between RCTs and non-randomized studies of similar follow-up duration.

### Data extraction and analysis

A.D.A., K.C., W.J.K., and F.d.V. performed data extraction. J.P.H. performed meta-analysis and designed and carried out statistical methodology with contributions from H.Y.J., L.C., D.P.F., Z.I.W., and M.J.S.S. The statistical programming environment R with the metafor package was used for all statistical analysis. Random-effects meta-analyses were performed using the restricted maximum likelihood estimator. For ordinal categorical outcomes (NYHA), Agresti's generalized odds ratios (ORs) were first calculated for each trial before meta-analysis.^7^^,^^8^ Interactions between groups were assessed using a mixed effects meta-analytical model with the variable in question as a moderator. We used the *I*^2^ statistic to assess heterogeneity. Included RCTs were assessed using the Cochrane risk of bias tool.

## Results

**Figure 1:**
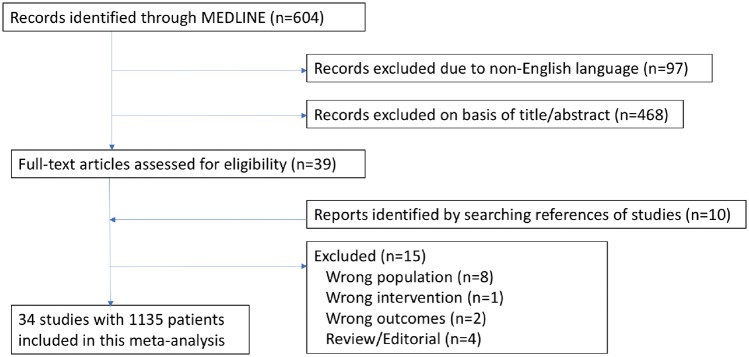
Flow chart for study selection.

We identified 604 study reports (flowchart in *Figure 1*), of which 34 were eligible for inclusion for at least one pre-specified outcome of interest, comprising 1135 patients.^6^^,^^9–40^ There were four RCTs,^20^^,^^24^^,^^28^^,^^31^ all blinded crossover trials, and 30 observational studies.^6^^,^^9–19^^,^^21–23^^,^^25–27^^,^^29^^,^^30^^,^^32–41^ Baseline characteristics are in *Table 1* and design features are in *Table 2*. Most studies reported data at multiple follow-up durations.^6^^,^^10^^,^^11^^,^^13–17^^,^^20^^,^^23^^,^^24^^,^^27^^,^^31^^,^^34–38^^,^^40–42^ Mean age was 55.5 years and mean baseline unpaced LVOTg was 78.9 mmHg.

**Table 1:** Characteristics of included studies—baseline values

Authors	Year	*N*	Age (years)	Male (%)	Baseline NYHA	Baseline LVEF (%)	Baseline LVOTg (mmHg)
Javidgonbadi *et al.*^a^	2017	88	55 ± 18	48	2.3 ± 0.6	69 (16)	64 (66)
Jurado Román *et al.*	2016	82	66 (range 22–88)	38	NA	73 ± 11	95 ± 37
Krejci *et al.*	2013	24	50 ± 17	NA	2.7 ± 0.5	70 ± 9	82 ± 46
Lucon *et al.*	2013	51	59 ± 14	47	2.7 ± 0.6	64 ± 8	79 ± 36
Yue-Cheng *et al.*	2013	37	52 ± 21	54	2.6 ± 0.8	64 ± 12	62 ± 11
Knyshov *et al.*	2013	49	38 ± 21	47	1.9 ± 0.8	NA	84 ± 15
Galve *et al.*	2010	50	62 ± 11	52	3.1 ± 0.3	76 ± 10	86 ± 29
Minami *et al.*	2010	24	52 ± 16	50	NA	NA	89 ± 38
Sandìn *et al.*	2009	72	64 ± 14	38	2.6 ± 0.5	67 ± 10	87 (IQR 61.5–115.2)
Binder *et al.*	2008	66	67	41	2.7 ± 0.7	NA	66 ± 36
Topilski *et al.*	2006	25	71 ± 12	48	3.2 ± 0.8	NA	92 ± 28
Hozumi *et al*.	2006	14	55 ± 16	79	NA	66 ± 6^b^	24 ± 12^b^
Megevand *et al*.^b^	2005	18	47	NA	2.4	NA	82 ± 35
Dimitrow *et al*.	2004	19	47 ± 16	52	3.2 ± 0.9	NA	77 ± 25
Mickelsen *et al.*	2004	11	69 ± 10	82	NA	NA	96 ± 21
Betocchi *et al.*	2002	21	45 ± 15	52	3.1 ± 0.4	NA	77 ± 37
Achterberg *et al.*	2002	7	52 ± 13	43	3.1 ± 0.5	NA	88 ± 13
Sant’Anna *et al.*	1999	9	47 ± 15	33	2.3 ± 0.5	NA	92 ± 22
Park *et al.*	1999	10	62 ± 13	50	3.5 ± 0.5	NA	83 ± 44
Sakai *et al.*	1999	12	55 ± 8	58	2.3 ± 0.5	NA	106 ± 47
Maron *et al.*	1999	44	53 ± 17	46	NA	NA	82 ± 33
Pak *et al.*	1998	5	48 ± 10	60	3	81 ± 8	67 ± 33
Simantirakis *et al.*	1998	8	56 ± 7	63	NA	NA	70 ± 18
Nishimura *et al.*	1997	19	59 ± 13	53	2.9 ± 0.4	NA	76 ± 61^c^
Gadler *et al*.	1997	22	68 ± 14	27	3 ± 0.6	NA	86 ± 40
Kappenberger *et al.*	1997	83	53 (range 32–87)	60	2.6 ± 0.5	NA	70 ± 24
Slade *et al.*	1996	52	48 ± 18	61	2.7 ± 0.6	NA	78 ± 31
Nishimura *et al*.^d^	1996	21	58 ± 16	50	NA	NA	73 ± 45
Gadler *et al.*	1996	22	65 ± 12	47	2.9 ± 0.6	NA	96 ± 33
Fananapazir *et al.*	1994	84	49 ± 16	50	3.2 ± 0.5	NA	96 ± 41
McAreavey *et al.*	1992	18	48 ± 14	44	3.3 ± 0.5	NA	94 ± 47
Jeanrenaud *et al.*	1992	13	56 ± 14	69	NA	NA	82 ± 41
Fananapazir *et al.*	1992	44	49 ± 14	50	3.4 ± 0.5	NA	64 ± 7
McDonald *et al.*	1988	11	51 ± 15	55	3 ± 0.6	NA	43 ± 25

Values for age, NYHA, EF, and LVOTg are mean ± standard deviation unless otherwise stated. Values for male are percentages. NA if not reported.

NYHA, New York Heart Association class; LVEF, left ventricular ejection fraction; LVOTg, left ventricular outflow tract gradient.

a LVOTg and LVEF data for this trial are reported as median (interquartile range).

b Baseline LVEF and LVOTg in this study is immediately *after* pacing is switched off after period of pacing (rather than prior to pacing initiation as performed in the other studies).

c Value reported from echocardiogram; 87 ± 54 on cardiac catheterization.

d This study contains the data from the acute haemodynamic protocol of Nishimura *et al*.^31^

**Table 2:** Characteristics of included studies—study design

Authors	Year	Study type	Optimal AV delay selection description	Optimal AV delay methods	Longest follow-up	AV delay (ms)
Javidgonbadi *et al.*	2017	Observational single-arm (retrospective)	‘set under ECG control to ensure abolition of spontaneous conduction and then evaluated by echocardiography to obtain maximal LVOT gradient reduction without deterioration of diastolic filling’	ECG Full capture TTE LVOTg Mitral filling pattern	16 ± 8 years	NA
Jurado Román *et al.*	2016	Observational single-arm (retrospective)	‘highest LVOTg reduction without excessive shortening of the ventricular filling time, as indicated by the minimal deterioration in the qualitative morphology of the mitral filling pattern on echocardiography’	TTE LVOTg Mitral filling	Median 8.5 years (range 1–18 years)	120 ± 16
Krejci *et al.*	2013	Observational signal-arm (retrospective)	‘set under ECG control to ensure full capture stimulation without the presence of spontaneous or fused contractions. In most patients, AV intervals were optimized under echocardiographic control so that LVOTg was reduced, while the stroke volume was not significantly affected’	ECG Full capture TTE LVOTg Stroke Volume	101 ± 49 months	NA
Lucon *et al.*	2013	Observational single-arm (retrospective)	‘longest interval associated with complete ventricular capture, at rest and during exercise. Radiofrequency modification of the AV junction was performed in patients whose short spontaneous PR interval precluded the complete capture of the ventricles. In patients whose P wave duration was ≥120 ms, a third lead was placed in the coronary sinus and connected to a biatrial DDD pacemaker to resynchronize the atria’	ECG Full capture Rest and exercise Invasive AVNA biatrial pacing	11.5 years (range 0.4–21.8)	NA
Yue-Cheng *et al.*	2013	Observational single-arm (retrospective)	‘AV delay (was) adjusted to 90–180 ms in order to ensure the ratio of ventricular pacing was more than 98%’	Device Full capture	4 years	120 ± 21
Knyshov *et al.*	2013	Observational single-arm (retrospective)	‘acute haemodynamic study with the real-time direct measurement of LVOTg during temporary pacing test in AAI, VDD, and DDD modes with different AV delays’	Invasive LVOTg^a^	68 ± 6.6 months	Range 45–120 (s), 85–180 (p)
Galve *et al.*	2010	Observational single-arm (prospective)	‘The optimal AV interval was defined as that obtaining a complete ventricular capture both at rest and during exercise’	ECG/device Full capture (rest and exercise)	5 ± 2.9 years	NA
Minami *et al.*	2010	Observational single-arm (prospective)	‘producing the lowest LVOTg without compromise of aortic pressure’	Invasive LVOTg, Aorta	Immediate	70 ± 30
Sandìn *et al.*	2009	Observational single-arm (retrospective)	‘method consisted of modifying the AV pacing interval and assessing the appearance of acute changes in the LVOTg, as well as the transmitral filling curves. The curve that achieved the largest gradient decrease without excessive shortening of the filling time was chosen’	TTE LVOTg Mitral filling	>1 year^b^	NA
Binder *et al.*	2008	Observational single-arm (retrospective)	Not stated	NA	3.7 ± 3 years	NA
Topilski *et al.*	2006	Observational single-arm (prospective)	‘To determine the optimized AVI, the AVI was set at 50 ms less than the native PR interval and increased in 25-ms steps, with five different AVIs. The AVIs were tested during sinus rhythm (VDD) and atrial pacing rates of 60 and 80 b.p.m. (DDD). At each AVI and heart rate combination, the LVOT gradient was measured. To achieve haemodynamic steady state, 15 min elapsed between pacemaker programming and the measurement of LVOTg. Systolic cuff blood pressure was used as an estimate of peak systolic aortic pressure. The AVI was programmed at the value with minimal LVOTg not associated with systolic arterial pressure reduction.’	TTE LVOTg Non-invasive: cuff BP	68 ± 34 months	106 ± 30
Hozumi *et al.*	2006	Observational single-arm (prospective)	‘optimized in individual patients to achieve the lowest LVOTg’	TTE LVOTg	7.4 ± 2.1 years	120 ± 31
Megevand *et al.*^c^	2005	Observational single-arm (prospective)	‘the longest interval that captured the ventricle and induced the greatest reduction in outflow gradient without compromising haemodynamics (during cardiac catheterization)’	Invasive LVOTg, haemodynamics	4.1 years (range 1–10)	Median 60
Dimitrow *et al.*	2004	Observational single-arm (prospective)	‘insure fully paced ventricular activation’	ECG Full capture	6 months	NA
Mickelsen *et al.*	2004	Randomized single-blinded crossover trial	‘DDD with an AV interval at 30 ms (DDD30)’^d^	Device Fixed 30 ms	1 month	30
Betocchi *et al.*	2002	Cohort (prospective non-randomized controlled observational study)	‘Italian cohort: AV interval was chosen as the one associated with the smallest gradient without a decrease in systolic blood pressure. UK cohort: AV interval was chosen as the one associated with the largest width of the QRS complex on the electrocardiograms’	TTE LVOTg BP ECG Full capture	1 year	89 ± 16
Achterberg *et al.*	2002	Observational single-arm (prospective)	‘programmed between 50 ms and 100 ms to ensure continuous ventricular capture’	Device Fixed 50–100ms	2.3 ± 1.1 years	50–100
Sant’Anna *et al.*	1999	Observational single-arm (prospective)	‘lowest LVOTg’	TTE LVOTg	6 months	NA
Park *et al.*	1999	Observational single-arm (prospective)	‘echocardiographic guidance to obtain maximal reduction in LVOTg’	TTE LVOTg	12 ± 11 months	82 ± 17 (s), 93 ± 19 (p)
Sakai *et al.*	1999	Observational single-arm (prospective)	‘produced the minimum LVOTg’	Invasive LVOTg, Aorta	1 year	NA
Maron *et al.*	1999	Randomized double-blinded crossover trial	‘longest interval which captured the ventricle and induced greatest reduction in LVOTg without compromising haemodynamics (i.e. decreasing blood pressure 30 mmHg), after testing a range of AV intervals’	Invasive LVOTg	1 year^g^	85 ± 35 (s)
Pak *et al.*	1998	Observational single-arm (prospective)	‘longest value that still yielded optimal ventricular pre-excitation as judged by QRS duration’	ECG Full capture	Immediate	NA
Simantirakis *et al.*	1998	Observational single-arm (prospective)	‘longest AV delay that produced a QRS complex of the same width as that seen in VVI pacing’	ECG Full capture	1 year	80 ± 23 (s)
Nishimura *et al.*	1997	Randomized double-blinded crossover trial	‘producing the lowest left ventricular outflow tract gradient without a significant decrease in aortic pressure or increase in left atrial pressure’	Invasive LVOTg, BP, LAP	3 months	71 ± 21
Gadler *et al.*	1997	Observational single-arm (prospective)	‘most pronounced reduction of left ventricular outflow tract gradient without any decrease in total mitral flow’	TTE LVOTg Mitral filling pattern	12 ± 9 months	64 ± 17 (s)
Kappenberger *et al.*^e^	1997	Randomized double-blinded crossover trial	‘full ventricular capture on the ECG without a drop in aortic pressure’	ECG Full capture Invasive	3 months	61 ± 23 (s)
Slade *et al.*	1996	Observational single-arm (prospective)	‘Shortest sensed AV delay not associated with haemodynamic deterioration, defined as a reduction in mean aortic pressure or cardiac output of >10%’	Invasive BP	11 ± 11 months	Median 65 (range 25–125) (s)
Nishimura *et al.*	1996	Observational single-arm (prospective)	‘longest AV interval in which there is full ventricular activation by the pacemaker without fusion complexes on the ECG’	ECG Full capture	Immediate	NA
Gadler *et al.*	1996	Observational single-arm (prospective)	‘resulting in the greatest reduction of LVOTg without reducing the integral of the A and E waves’	TTE LVOTg Mitral filling pattern	Immediate	60–80 (s)
Fananapazir *et al.*^f^	1994	Observational single-arm (prospective)	‘longest interval that permitted ventricular pre-excitation (maximum widening of the QRS complex during the exercise tests)’	ECG Full capture (exercise)	2.3 ± 0.8 years	120 ± 9 (s)
McAreavey *et al.*	1992	Observational single-arm (prospective)	Not stated	NA	12 weeks	NA
Jeanrenaud *et al.*	1992	Observational single-arm (prospective)	‘best reduction in LVOTg without drop in mean aortic pressure’	Invasive LVOTg	44 ± 11	63 ± 18
Fananapazir *et al.*	1992	Observational single-arm (prospective)	‘longest AV delay that allowed for maximal pre-excitation (widest-paced QRS duration)’	ECG Full capture	1.5–3 months	115 ± 17 (s)
McDonald *et al.*	1988	Observational single-arm (prospective)	‘highest value that maintained ventricular capture at maximum exercise’	ECG Full capture (exercise)	1 h	90 (range 50–150)

Values are represented as mean ± standard deviation unless otherwise stated. Study type refers to arms of trials fulfilling inclusion criteria. Single-arm studies refer to studies where there is either one arm or where other arms are not control groups with conventional/medical/non-interventional therapy (instead they are alternative therapies). Optimal AV delay selection is the description of optimal AV delay selection in the wording of the source text with changes to wording made only to paraphrase and abbreviate. Optimal AV delay methods refer to the techniques used in determining the optimal AV delay. AV delay refers to the AV delay used in the trial; where the AV delay is specifically stated to be sensed AV delay, this is acknowledged by (s), and where paced (p).

a Unclear from publication whether invasive or echocardiographic but wording suggests invasive measurement.

b Precise duration of follow-up not stated but longer than 1 year.

c Non-responders in initial acute study did not undergo implantation of pacemaker.

d This study also included an arm where pacing was optimised to peak aortic flow but LVOTg response to this was not reported for all patients (only for responders and non-responders).

e Long-term follow-up data.

f Long-term follow-up of Fananapazir *et al.*^13^

g Crossover periods were for 3 months but after the crossover study 6 months of DDD pacing occurred.

### Risk of bias

Trial quality for the four RCTs, assessed by the Cochrane risk of bias assessment tool, is shown in *Table 3*. Three were rated as low risk of bias due to double blinding but the fourth was single blinded. Lack of randomization and lack of blinding put all 31 observational studies at risk of bias.

**Table 3:** Risk of bias assessment—randomized controlled trials

Authors	Random sequence generation	Allocation concealment	Blinding of participants and personnel	Blinding of outcome assessment	Incomplete outcome data	Selective reporting	Overall quality
Kappenberger *et al.*	Low risk	Low risk	Low risk	Low risk	Low risk	Low risk	Low risk
Nishimura *et al.*	Uncertain	Uncertain	Low risk	Low risk	Low risk	Low risk	Low risk
Maron *et al.*	Uncertain	Uncertain	Low risk	Low risk	Low risk	Low risk	Low risk
Mickelsen *et al.*	Uncertain	Uncertain	High risk	High risk	Low risk	Low risk	High risk

### Left ventricular outflow tract obstruction

**Figure 2:**
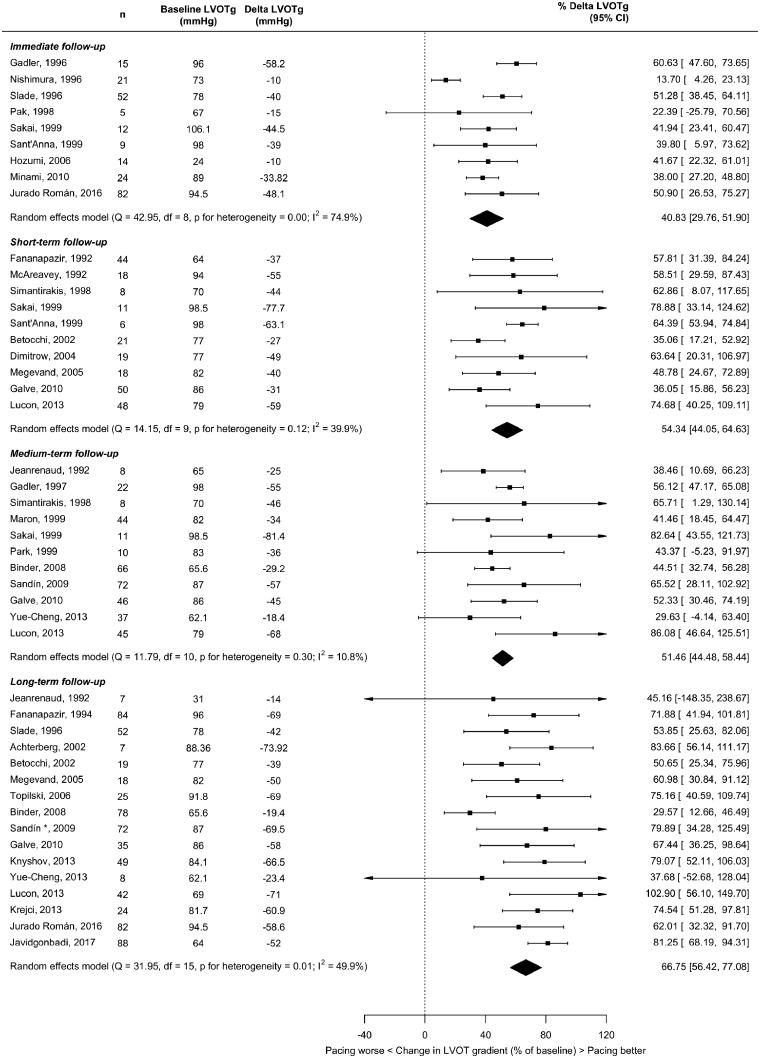
Effect of right ventricular pacing on left ventricular outflow tract gradient at immediate (<12 h), short-term (12 h to 6 months), medium-term (>6 months to <2 years), and long-term (at least 2 years) follow-up in non-randomized studies.

**Figure 3:**
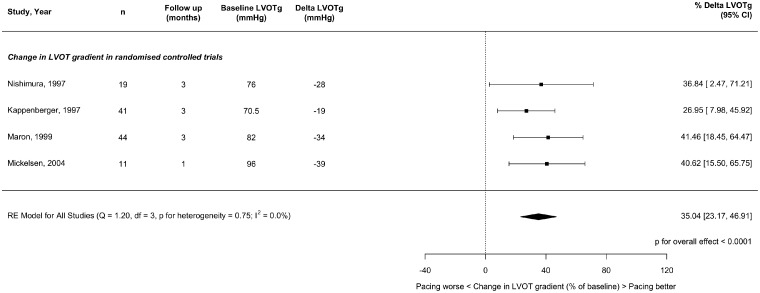
Effect of right ventricular pacing on left ventricular outflow tract gradient at short-term follow-up (1–3 months) in crossover randomized controlled trials.

Thirty-two studies reported mean change in LVOTg (ΔLVOTg) from baseline for at least one follow-up duration. The results are shown in *Figure 2* for observational studies, *Figure 3* for RCTs, and summarized in *Table 4*.

**Table 4:** Results

Trials (*n*)	Patients (*n*)	Study type^a^	Follow-up duration	Pooled result	95% confidence interval	*P*-value	*I* ^2^ heterogeneity	*P*-value for heterogeneity
Percentage change in LVOT gradient from baseline								
9	234	Obs	Immediate (<12 h)	−40.8%	−29.8 to −51.9	<0.0001	74.9% (high)	<0.0001
10	243	Obs	Short-term (12 h to 6 months)	−54.3%	−44.1 to −64.6	<0.0001	39.9% (moderate)	0.12
11	369	Obs	Medium-term (>6 months to <2 years)	−51.5%	−44.5 to −58.4	<0.0001	10.8% (low)	0.3
16	644	Obs	Long-term (at least 2 years)	−66.8%	−56.4 to −77.1	<0.0001	49.9% (moderate)	0.01
4	115	RCT	Short-term (1–3 months)	−35%	−23.2 to 46.9	<0.0001	0% (low)	0.75
Odds ratio for improved NYHA class from baseline								
9	388	Obs	All follow-up durations	8.39	4.39 to 16.04	<0.0001	74.9% (high)	<0.0001
3	137	RCT	All follow-up durations	1.82	0.96 to 3.44	0.066	81.7% (high)	0.0042

Obs, observational studies (non-randomized); RCT, randomized controlled crossover trials.

In the four RCTs (follow-up ranged from 1 months to 3 months), pacing reduced LVOTg by 35%. The observational studies reported slightly larger LVOTg reductions (*Table 4*).

Meta-regression showed progressively greater gradient reductions at longer follow-up durations, by an average of 5.2% per month [confidence interval (CI) 2.5–7.9, *P* = 0.0001].

We, therefore, compared gradient effect size of RCTs with observational studies at similar follow-up times (denoted short-term). The observational studies reported a gradient reduction, which was 18.6% greater than RCTs (CI 1.3–36, *P* = 0.0351).

### New York Heart Association status

**Figure 4:**
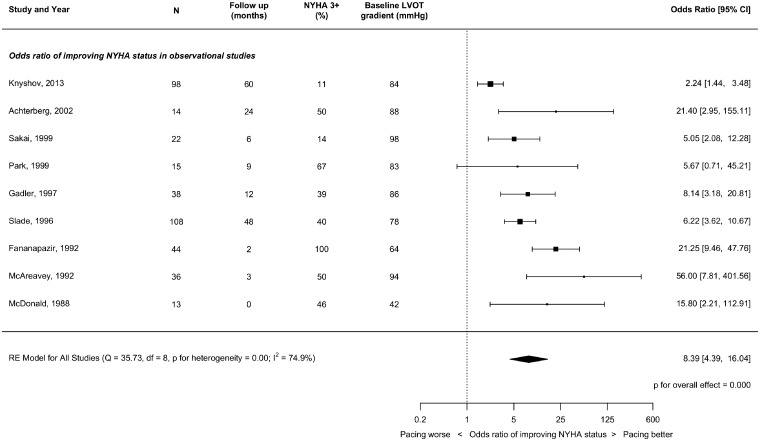
Effect of right ventricular pacing on New York Heart Association class at short-term follow-up (1–3 months) in non-randomized studies.

**Figure 5:**
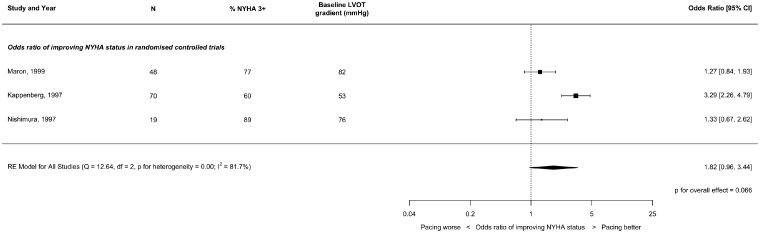
Effect of right ventricular pacing on New York Heart Association class at short-term follow-up (1–3 months) in crossover randomized controlled trials.

As pre-specified, because there were too few studies reporting NYHA, we did not sub-divide them by follow-up duration. The results are displayed in *Figures 4 and 5* and summarized in *Table 4*.

In RCTs, there was a trend towards improved NYHA class with pacing (OR 1.82, CI 0.96–3.44; *P* = 0.066) but with considerable heterogeneity (*I*^2^ = 81.7%) as a result of one trial showing a particularly prominent favourable effect.

In contrast, almost all of the observational studies (8/9) reported marked improvement in NYHA class giving a combined OR of 8.39 (CI 4.39–16.04, *P* < 0.0001). This OR is over 450% of the OR for RCTs: ratio of ORs 4.54 (CI 1.61–12.82, *P* = 0.0042).

### Exercise and systolic function

For each of the other variables (ejection fraction, exercise duration, and peak oxygen uptake) fewer than five studies reported the data require, and therefore, meta-analysis was not conducted. Reported changes in ejection fraction ranged from +3% to −11%, and in exercise duration from +0.3 min to +3.1 min. There was a single RCT report of peak oxygen uptake change, −0.1 mL/kg/min.

## Discussion

This is the first meta-analysis assessing the role of right ventricular pacing as a treatment for LVOTO in HOCM. We found that, in blinded RCTs, pacing reduces LVOTg and shows a non-significant trend to reduce NYHA class. Unblinded observational studies report very much larger symptomatic effects, suggesting an unintentional bias much larger than any genuine effect of the pacing. Echocardiographic gradient assessments, which appear less vulnerable to this bias, suggest a progressive enhancement of the therapeutic effect with the passage of time.

### Left ventricular outflow tract gradient reduction

The meta-analysed RCT data strongly supports the concept that pacing reduces measured LVOTg. There are broadly four mechanisms by which pacing can have an influence. Right ventricular pacing causes incoordination of ventricular activation which may reduce the driving force of ventricular ejection. The altered ventricular activation sequence attenuates the tendency of the left ventricular outflow tract (LVOT) lumen to become very small during systole. Atrioventricular (AV) sequential pacing alters ventricular filling through a change in AV delay, which impacts on ventricular ejection. Finally, knowledge that the patient is receiving a treatment believed to decrease LVOTg may cause an unintended bias of the echocardiographer to try less hard to find a high gradient.

Randomization with allocation concealment (‘blinding’) is the most effective approach to reduce unintended bias when evaluating therapies. Under blinded conditions each of the trials individually showed that pacing reduced gradient. The measured effect was much larger in the (unblinded) observational studies, −55% rather than −35% (*P* = 0.0351 for difference in study designs). While it is not certain that the much larger effect sizes reported by unblinded studies is due to unintended bias, it is difficult to imagine that the patient groups or procedural characteristics were so markedly different between the two study designs.

### Symptoms without blinding

The blinded RCTs showed an encouraging trend towards a statistically significant reduction in NYHA class. A very much larger reduction in NYHA class was reported by the unblinded observational studies. The pooled estimates were so far apart their CIs did not overlap. Indeed, the point estimates for eight out of nine observational studies were for a greater NYHA reduction than even the highest upper limit of the CI of any individual RCT.

We conclude from this that there may be a symptomatic benefit, but that unblinded study design provides no useful information on it. We cannot even use the unblinded symptomatic relief data to compare different studies in order to select pacing approaches for future blinded trials. This is because the great majority of the unblinded symptomatic relief appears to be bias. Therefore, the differences between the unblinded symptom-relief effects reported by different studies will be dominated by differences in the amount of bias rather than clinically meaningful differences in protocol.

The origin of bias in unblinded observational studies investigating subjective outcomes, such as NYHA, may be due to the anticipation of effect by patients. However, the clinicians that rate patients’ NYHA status, who are also aware of treatment allocation, may also contribute to bias with their own expectations of successful, or, indeed, unsuccessful, treatment.

### Clinical implications

Knowing whether AV sequential right ventricular pacing is beneficial in HOCM is important because many patients with HOCM require a defibrillator and the modern era offers two changes from the traditional dual-chamber, transvenous defibrillator. First, omitting the atrial lead can reduce complication rates^43^ but it prevents AV sequential pacing. Second, subcutaneous defibrillators are now available, which are easier to remove than trans-venous defibrillators, but do not have any pacing function.

If AV sequential right ventricular pacing genuinely helps patients, then dual chamber, transvenous defibrillators would be preferable over both single chamber transvenous and subcutaneous defibrillators. Although the blinded RCTs show only a non-significant trend to reduction in NYHA class, the point estimate of the pooled effect size is an OR of 1.82. Roughly speaking, this means patients are twice as likely to feel better with pacing switched on rather than off. This is a potentially meaningful clinical benefit and merits further investigation through further blinded RCTs. Such RCTs need not be expensive or resource-intensive, recruiting patients undergoing *de novo* implantation. They could be conducted in patients who already have a defibrillator, with two randomized periods (pacing on vs. off) and appropriately blinded evaluation. A 2012 Cochrane review^2^ of observational and randomized studies concluded, as we do, that the existing observational studies are of low quality due to inadequate blinding but they did not quantify this effect as we have done. They also call for high quality trials to investigate the potential for a true symptomatic benefit.

The clear evidence of gradient reduction by pacing should not be assumed to prove that symptoms also improve. This is because pacing (i) reduces the force of contraction by inducing incoordination, (ii) reduces the impingement of the LVOT during systole, and (iii) necessarily alters filling since the paced activation must begin before the native activation would have otherwise occurred. The symptomatic effect will therefore be a result of not only changes in LVOT calibre but also ventricular filling and ejection. The choice of AV delay may be important. Applying a very short AV delay almost always reduces stroke volume, even if systolic LVOT calibre is increased. It is unclear how to programme the AV delay of a pacemaker in a patient with HOCM, to provide a net advantage. Each of the studies has taken a different approach, which may have contributed to the lack of translation of gradient reduction to symptomatic benefit, and more work on this is required. The TRICHAMPION trial is underway, which is examining the role of AV nodal ablation, for complete control of AV delay, with biventricular pacing. Importantly, not all symptoms contributing to NYHA status in HCM are due to LVOTO, some maybe due to the cardiomyopathic process itself or comorbidities, and thus unaffected by pacing.

The meta-regression of gradient with respect to follow-up duration suggests a progressive enlargement of the therapeutic effect with longer follow-up across all published data, as has been noted before within single studies.^34^^,^^40^ It is not yet clear what the mechanism for such a progression might be. One possibility is progressive changes in the structure of the ventricle. However, it should be remembered that long-term right ventricular pacing is known to cause left ventricular function to deteriorate, and therefore, a long-term reduction in gradient should not automatically be assumed to be beneficial.

### Limitations

There were only four RCTs, although all were blinded. Studies mostly did not report lead position or baseline QRS morphology, preventing separate analysis of the apical and septal positions or the presence of pre-existing bundle branch block or meta-regression for these variables. The apical lead position may be expected to result in more dyssynchronous activation and thus greater ‘beneficial’ LVOTg reduction, but this may be offset, or even superceded, by dyssynchrony-related reduction in myocardial performance. This has importance in procedure technique as achieving an apical lead position in severe hypertrophy can be technically challenging.

Many studies reported the mean baseline (unpaced) and the mean follow-up (paced) LVOTg and NYHA class with a categorical description of the *P*-value (e.g. ‘*P* < 0.05’) for the statistical test for differences between them. The most useful value, however, would be the mean intra-patient change in LVOTg along with its CI, standard error, standard deviation, or precise *P*-value. This has limited the precision with which we can calculate the CI for the pooled estimate for the change in LVOTg and NYHA, but has not affected the point-estimate.

## Conclusions

The blinded RCTs show that AV sequential right ventricular pacing reduces LVOTg in HOCM, and shows a trend to benefit in symptoms that is not statistically significant but of a potentially clinically meaningful size. This may have implications for the choice of defibrillator to implant in HOCM. More research is needed into appropriate selection of AV delay. More blinded RCTs on symptom relief are required and they need not be very demanding or expensive.

Unblinded observational studies report substantially larger gradient reductions and very much larger symptom reductions. The difference between the effect sizes reported by the two study designs (RCT vs. observational) is so large that observational data are of uncertain value in progressing the field. This is particularly so for symptoms, where the unblinded effect sizes appear to be scaled up by 450%.

## Supplementary Material

qcz006_Supplementary_Materials
