# Supplementary material for: Right ventricular pacing for hypertrophic obstructive cardiomyopathy: meta-analysis and meta-regression of clinical trials
Source: Eur Heart J Qual Care Clin Outcomes. 2019 Jan 31;5(4):321–33. doi: 10.1093/ehjqcco/qcz006 (PMC6775860; doi:10.1093/ehjqcco/qcz006)
Supplement: qcz006_Supplementary_Materials [file qcz006_supplementary_materials.docx]

# Supplementary Materials

## Search Strategy

We searched for studies that included one of the following terms in titles and abstracts:

*Hypertrophic Cardiomyopathy (including as MeSH term)*

*Left Ventricular Outflow Tract Obstruction*

*Left Ventricular Hypertrophy Gradient*

*Left Ventricular Hypertrophy*

Along with one of the following terms in titles and abstracts:

*Right Ventricular Pacing*

*Pacing*

*Pacemaker*

*Ventricular Pacing*

The following search was performed in PubMed:

*"Search ((((((""Hypertrophic Cardiomyopathy""[Title/Abstract]) OR ""Hypertrophic Cardiomyopathy""[MeSH Terms]) OR ""Left Ventricular Outflow Tract Obstruction""[Title/Abstract]) OR ""Left Ventricular Outflow Tract Gradient""[Title/Abstract]) OR ""Left Ventricular Hypertrophy""[Title/Abstract])) AND ((((""Right Ventricular Pacing""[Title/Abstract]) OR ""Pacing""[Title/Abstract]) OR ""Pacemaker""[Title/Abstract]) OR ""Ventricular pacing""[Title/Abstract])"*

## Detailed Endpoint Criteria

The primary efficacy endpoint was LVOT gradient reduction. Trials were included if they reported the mean change in LVOT gradient (ΔLVOTg) from baseline (unpaced) to follow up (paced) and the standard deviation (SD), standard error (SE) or p value for the significance test of this change. Trials were also included if they provided the baseline and follow up mean values, without reporting the mean change, as long as the p value of the significance test from baseline to follow up was reported. If the upper limit of the p value (eg p<0.001 rather than p=0.0006) was reported this was used to determine the widest confidence interval surrounding the ΔLVOTg. It was prospectively determined that ΔLVOTg was to be measured as a percentage change from baseline value rather than absolute change in mmHg.

The secondary efficacy outcomes were change in symptomatic and functional status as measured by NYHA status, exercise time and peak oxygen uptake, from baseline to follow up. For ΔNYHA, trials were included if the raw data for NYHA status at baseline and follow up were included, where the median or mean for baseline and follow up or the change were reported along with the actual or upper limit of the p value for significance testing. EF, ED and mVO_2_ estimates would be pooled if at least five studies reported their change with pacing from baseline with sufficient variance data (SD, SE p value for change from baseline). Since it was anticipated that that these outcomes would be less frequently reported, if fewer than five studies reported this adequately a descriptive analysis would be undertaken.
